# Xanthogranuloma of the Sellar Region: A Comprehensive Review of Neuroimaging in a Rare Inflammatory Entity

**DOI:** 10.3390/jpm12060943

**Published:** 2022-06-08

**Authors:** Vera Lozovanu, Carmen Emanuela Georgescu, Lavinia Maria Florescu, Carmen Georgiu, Horatiu Silaghi, Andrian Fratea, Cristina Alina Silaghi

**Affiliations:** 1 Department of Endocrinology, “Iuliu Hatieganu” University of Medicine and Pharmacy Cluj-Napoca, 8 Victor Babes Street, 400012 Cluj-Napoca, Romania; lozovanu.vera9@gmail.com (V.L.); alinasilaghi@yahoo.com (C.A.S.); 2 County Clinical Emergency Hospital Cluj, 3-5 Clinicilor Street, 400006 Cluj-Napoca, Romania; florescumarialavinia@gmail.com; 3 Department of Pathological Anatomy, “Iuliu Hațieganu” University of Medicine and Pharmacy Cluj-Napoca, 8 Victor Babes Street, 400012 Cluj-Napoca, Romania; carmengeorgiu@hotmail.com; 4 Department of Surgery V, “Iuliu Hatieganu” University of Medicine and Pharmacy Cluj-Napoca, 8 Victor Babes Street, 400012 Cluj-Napoca, Romania; hsilaghi@yahoo.com; 5 Clinical Emergency Pediatric Hospital, 68 Moților Street, 400370 Cluj-Napoca, Romania; adrianfratea@yahoo.com

**Keywords:** xanthogranuloma, chronic inflammation, sellar region, MRI, neuroimagistics, cystic, craniopharyngioma, Rathke’s cleft cyst, pituitary adenoma

## Abstract

Xanthogranuloma of the sellar region is a rare chronic inflammatory lesion resulting from secondary hemorrhage, inflammation, infarction, and necrosis of an existing Rathke’s cleft cyst, craniopharyngioma, or pituitary adenoma. Sellar xanthogranulomas are challenging to differentiate from other cystic lesions preoperatively due to the lack of characteristic imaging features. We performed a literature overview of the clinical and paraclinical features, treatment options, and long-term outcomes of patients with sellar xanthogranuloma, focusing on the preoperative radiological diagnosis. The hyperintense signal in both T1- and T2-weighted sequences, cystic or partially cystic morphology, ovoid shape, sellar epicenter, intra- and suprasellar location, intratumoral calcifications, linear rim contrast enhancement, and the absence of cavernous sinus invasion suggest xanthogranuloma in the preoperative differential diagnosis. An endoscopic endonasal gross total resection without radiotherapy is the preferred first-line treatment. Given the low rate of recurrence rate and low chance of endocrinological recovery, a mass reduction with decompression of the optic apparatus may represent an appropriate surgical goal. Identifying the xanthogranulomas’ mutational profile could complement histopathological diagnosis and give insight into their histo-pathogenesis. A better preoperative neuroimagistic diagnosis of sellar xanthogranulomas and differentiation from lesions with a poorer prognosis, such as craniopharyngioma, would result in an optimal personalized surgical approach.

## 1. Introduction

Xanthogranulomas (XGs), also known as cholesterol granulomas, are rare and poorly understood pathological entities with a 1.6–7% incidence of autopsies [1]. They can occur at various sites inside and outside the cranial vault, and more frequently involve the middle ear, the mastoid bone, and the paranasal sinuses [2,3,4,5]. Intracranial XGs arise predominantly in the choroid plexus, and a few are found in the cerebral parenchyma, or the third or fourth ventricle [6,7,8], whereas those that occur in the sellar region are extremely rare [3]. However, sellar and juxta-sellar XGs are considered distinct entities.

XG was first reported in 1988 in only 4 of 211 sellar tumors [9]. They were conventionally classified as a variant of adamantinomatous craniopharyngiomas (aCPs), as they both share cholesterol clefts, even in the absence of epithelial tissue [9]. It was then hypothesized that XGs are degenerative masses induced by chronic inflammation in the circumscribed area of a CP [1,5,10]. In 1999, Paulus et al. described 37 of 100 craniopharyngiomas (CPs) with massive cholesterol reaction, non-adamantionomatous squamous epithelium, or missing epithelium, a predominant intrasellar location, and favorable outcome [3]. These findings suggested that xanthogranulomatous lesions are histologically and clinically distinct from aCPs [3]. In 2000, XG of the sellar region was added as a new entity to the World Health Organization brain tumor classification [11].

**Figure 1:**
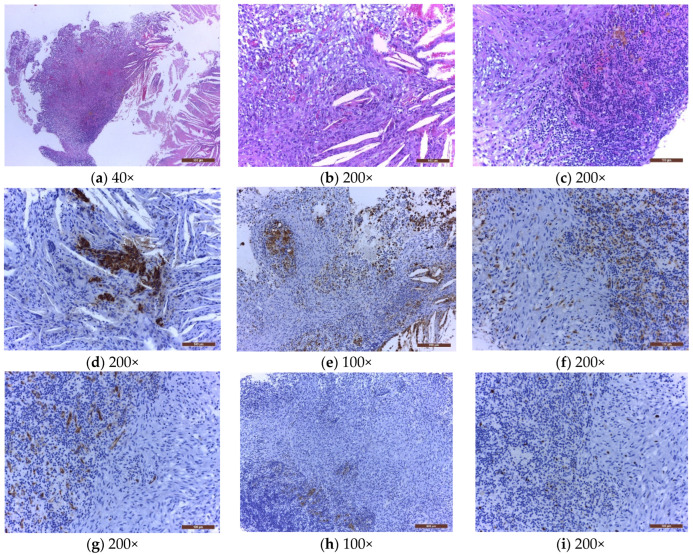
The microscopical examination of a sellar xanthogranuloma (**a**) solid area formed by a mixture of tumoral/inflammatory cells, and clear acicular cholesterol crystals, at low magnification (40×); (**b**) mixture of foam cells, spindle cells, rare lymphocytes, and cholesterol crystals with foreign body multinucleated giant cells around them (200×); (**c**) mixture of spindle cells, large number of lymphocytes, plasma cells, erythrocytes, golden brown siderophages, and small vessels (200×); (**d**) cholesterol crystals and a few remnants of dark brown pituitary glandular cells, positive for chromogranin A; (**e**) dark brown macrophages (foam cells, histiocytes, some spindle cells, and multinucleated giant cells), positive for CD 68 (100×); (**f**) dark brown macrophages (histiocytes and some spindle cells), positive for Factor XIIIa (200×); (**g**) small vessels with dark brown endothelial cells, positive for CD34 (200×); (**h**) spindle cells negative for TTF1 (100×); (**i**) low Ki67 expression in spindle cells and lymphocytes (200×). Hematoxylin and eosin (**a**–**c**) and immunohistochemistry (**d**–**i**). The nature of the spindle cells raised several differential diagnoses. Immunohistochemically, regardless of their type (histiocytes, foam cells, siderophages, foreign body giant cells), all the macrophages were CD68 and Factor XIIIa positives (Figure 1e,f). However, some of the spindle cells were also CD68 and Factor XIIIa positives, raising the possibility of their monocytic/macrophagic nature. In the evolution of the xanthogranulomas, the macrophages may transform, the spindle cells representing the most aged form of the spectrum of these cells. This diagnosis was also reinforced by the fact that the spindle cells were immunohistochemically negative to CD34 (Figure 1g), excluding a fibroblastic nature; negative to TTF1 (Figure 1h), excluding a pituicytoma; negative to S100, excluding a Langerhans cell histiocytosis; negative to BRAFV600E, excluding an Erdheim Chester disease or a more aggressive juvenile xanthogranuloma; and with a very low Ki67 proliferation index (Figure 1i).

Histologically, XG results from a granulomatous reaction, characterized by cholesterol clefts, hemosiderin deposits, accumulation of multinucleated foreign body giant cells and foamy macrophage, necrotic debris, and fibrous proliferation [3], as illustrated in Figure 1. 

Prior to surgery, XGs are difficult to distinguish from other cystic lesions of the sellar region, such as CPs, Rathke’s cleft cyst (RCCs), pituitary adenomas (PAs), arachnoid cysts, epidermoid cysts, and dermoid cysts [12,13], due to the paucity of sellar XGs cases reported in the literature and the lack of characteristic imaging features. 

Moreover, both the diagnosis and definition of surgical goals for sellar XGs continue to be challenging. Compared to CPs and RCCs, sellar XGs show a lower recurrence rate, even after subtotal tumor removal (STR). Moreover, XGs cause severe multi-axial endocrine deficits prone to remain postoperatively, and visual field disturbances which, in turn, show significant postoperative improvement [11,13,14,15]. Therefore, a more precise preoperative diagnosis would guide the surgical team towards a more conservative approach, namely tumor mass reduction with decompression of the optic apparatus in cases highly suggestive for XGs. Herein, we aimed to review the literature comprehensively and discuss the clinical, imagistic features and outcomes of patients with sellar XGs, focusing on radiological findings that can contribute to a more accurate preoperative diagnosis. In the light of recent advances in genetics, we also aimed to gather all the available data about the molecular profile of sellar XGs.

## 2. Histogenesis and Pathogenesis

**Figure 2:**
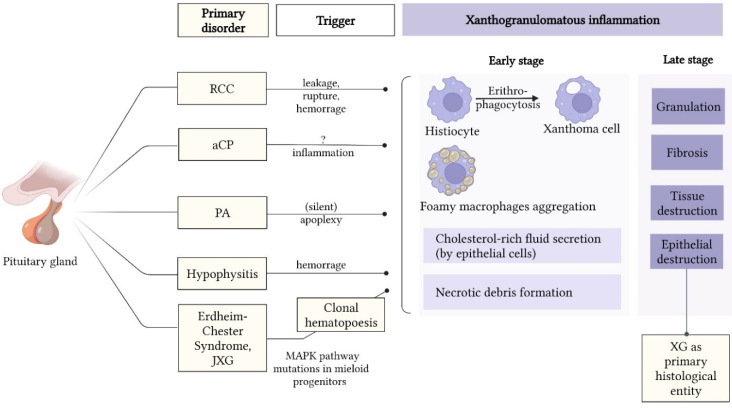
Theories explaining the etiopathology of sellar XG. Abbreviations: aCP = adamantinomatous craniopharyngioma; CP = craniopharyngioma; JXG = juvenile xanthogranuloma; MAPK = mitogen-activated protein kinase; PA = pituitary adenoma; RCC = Rathke’s cleft cyst; XG = xanthogranuloma.

Nowadays, the etiology and pathogenesis of sellar XGs remain unclear and probably heterogeneous. Several conflicting theories aim to explain their origin (Figure 2). The most frequently postulated is that XG is the late stage of a secondary response to repeated inflammation, bleeding, or degeneration of an RCC or CP [16]. 

RCCs, CPs, and colloid cysts may develop a severe inflammatory reaction triggered by cyst fluid leakage into the surrounding tissue or intratumoral hemorrhage, leading to secondary granulomatous degeneration [11,13,17]. Hemosiderin is a ubiquitous component of XGs, proving that hemorrhages are an essential chain [18,19]. The cause of RCC hemorrhage is unknown, despite the absence of a relevant history of trauma, apoplectic episodes, or severe headache in these patients [20,21]. The origin of xanthoma cells is poorly described in the literature, but they probably arise from the histiocytes in close contact with hemorrhagic foci after engulfing erythrocytes [19].

XG is often a component within other tumors such as RCCs, CPs, and PAs [20]. Cuboidal or columnar epithelium, as well as squamous metaplasia, characteristic of RCC, have been found in 86% (6/7 cases) and 67% (4/6 cases) of sellar tumors, consisting primarily of xanthogranulomatous material [11,22]. This feature suggests that RCCs could be the most frequent underlying substrate in XGs development. There is also a subset of XGs without epithelium, whose etiology cannot be determined. It was hypothesized that inflammation and bleeding could destroy epithelial cells, leaving only xanthogranuloma tissue. Besides, incomplete surgical excision has been suggested to exclude epithelial elements from the excised specimen [3,11,17]. Interestingly, no papillary CP with secondary xanthogranulomatous change was found [20]. 

Xanthogranulomatous changes in PAs result from a secondary degenerative process, probably following silent apoplexy [21]. A few cases of xanthogranulomatous PAs are reported in the literature [23,24,25]. Nishioka et al. found that 5 (2.2%) of 231 nonfunctional pituitary macroadenomas treated by surgery were associated with xanthogranulomatous reaction [21]. 

Besides, extreme adenohypophysitis and infundibuloneuro-hypophysitis may act as a trigger for xanthogranulomatous reactions [26]. XG represents a spectrum of xanthomatous inflammation together with xanthomatous and xanthogranulomatous hypophysitis (XH), with multiple cases showing foci that fall into more than one of these categories [18]. Histologically, XH is characterized by infiltration of foamy xanthoma cells and lymphocytes, resulting from macrophage activation, multinucleated giant cells, and epithelioid histiocytes [18,27,28]. The progressive accumulation of hemosiderin pigment in the XH, presumably due to multiple bleeding episodes, could account for the transition of some XHs to XGs [18]. It is suggested that only XHs with massive or repeated hemorrhage develop giant cell reactions around the extensive cholesterol cleft formation and substantial hemosiderin deposits [18]. The overlap between XHs and XGs can explain the origin of a minority of XGs that cannot be linked to RCC leakage, rupture, or hemorrhage [18].

XGs of the sella may also be associated with systemic diseases such as juvenile XG (JXG), Erdheim–Chester disease (ECD), Hand–Christian syndrome, tuberculosis, or sarcoidosis [29,30,31].

JXG is a non-Langerhans histiocytic proliferation, primarily affecting children. Immunohistochemistry and histopathology are used to diagnose JXGs, as they are positive for CD14, CD68, CD163, factor XIII, and fascin, and negative for CD1a CD207, S110, and langerin [32,33]. The development of JXG is poorly understood. However, mutations in the mitogen-activated protein kinase (MAPK) pathway genes linked to JXG, such as *KRAS*, *NRAS*, *ARAF*, *MAP2K1*, *PIK3CD*, and *BRAFV600E*, have been identified [34,35,36]. ECD is an uncommon histiocytic condition that appears to be a member of the JXG family morphologically and immunohistochemically [33]. In samples from ECD patients, recurrent somatic *BRAFV600E* mutations have been identified [37]. Other MAPK pathway-activating mutations, such as *NRAS*, *RAS*, *RAF*, *MAPK-ERK*, and *PI3K-AKT*, have been described [38]. The cases of XGs within systemic diseases have not been discussed in the current review. 

## 3. Demographics and Clinical Presentation

The reported prevalence of XGs among sellar and para-sellar tumors varies between 0.6–3% [15,17,22]. XGs are generally present in the second and fourth decades of life [39]. In the largest cohorts, a slight male predominance has been identified [3,5,25,40]; however, smaller studies have opposite findings [17,18,22,41].

The chief complaints of patients with sellar XGs reported by Vasques et al. in a review of 32 cases were headache (53%), visual disturbances (22%), and symptoms related to endocrine deficits [25]. In 53% of cases, panhypopituitarism was the most frequent endocrinological abnormality, whereas normal pituitary function was found in 21.9% [25]. Hernández-Estrada et al., in a review of 30 cases, found that endocrinological deficit was the most common presentation, manifested as either panhypopituitarism or diabetes insipidus. Amano et al. reported that 86% of patients with sellar XGs presented with anterior pituitary deficiency involving 2–6 axes. 

Vasquez et al. noted hyperprolactinemia in 12.5% of the included patients. In most cases, the stalk effect could explain hyperprolactinemia [25]. However, a single case report was published with elevated serum prolactin due to a prolactinoma with secondary xanthogranulomatous changes [25]. Sporadic cases were manifested as obstructive hydrocephalus with acute changes in consciousness [39].

Cases of XGs in the pediatric population are rare and difficult to discriminate from CPs or RCCs. In a review of 17 cases of XGs, the median age at diagnosis was 10 years (range, 5–15), without evidence of sex distribution. As in adults, the most common initial symptom of XGs in children was headache (47%) [42]. By contrast, polyuria, polydipsia (29%), and growth retardation (18%) were the second and third most common symptoms in children [42]. Less frequent symptoms were loss of appetite, amenorrhea, blurred vision, and homonymous hemianopsia. Preoperative visual disturbances were found in 41% of cases. 

## 4. Neuroimaging of Sellar Xanthogranulomas

### 4.1. Demographics in the Reviewed Cases of Sellar Xanthogranuloma

PubMed was employed as the search engine, using the following keywords: “xanthogranuloma” or “cholesterol granuloma” and “sellar region”. Articles were included if they presented a formal description of magnetic resonance imaging (MRI) appearance and the postoperative histopathology of the tumors. XGs not primarily involving the sellar and para-sellar region (petrous apex), JXGs, and XGs belonging to the ECD or Rosai–Dorfman Syndrome and XHs were excluded. Additional exclusion criteria included articles written in languages other than English, Spanish, or German. Two reviewers independently conducted data extraction from the articles’ text, tables, and MR images.

We identified 29 articles published between 2003 to 2021, reporting a total of 50 patients with the diagnosis of sellar or juxta-sellar XGs who underwent MRI preoperatively (Table 1). Patients’ age ranged from 5 months to 73 years old (mean age, 41.5; median age, 36.5). The cohort had a uniform sex distribution with a slight male predominance, including 26 males and 23 women. For one patient, the data about gender were not found.

### 4.2. Sellar Xanthogranulomas MRI Pattern of Morphology

The most common morphology pattern of the revised cases was the cystic lesion (n = 20), characterized by a hyperintense signal on both T1WI and T2WI sequences, and no or faint contrast enhancement. In five cases, the content of the cyst was homogenously hyperintense in both T1WI and T2WI, whereas the other 15 cysts had a heterogeneous composition, mainly hyperintense with small hyperintense areas in T1WI and hypointense in T2WI, or hypointense areas in both sequences. 

A solid morphology pattern was noted in 14 tumors, primarily characterized by inhomogeneous content, occasionally suggesting subacute bleeding. The solid masses appeared heterogeneously hyper- or isointense in T1WI, and hyper-, iso-, or hypointense in T2WI. Most XGs showed an inhomogeneous contrast enhancement, although a minority of cases presented no contrast enhancement [15,42] or a thin peripheral LR [1].

**Figure 3:**
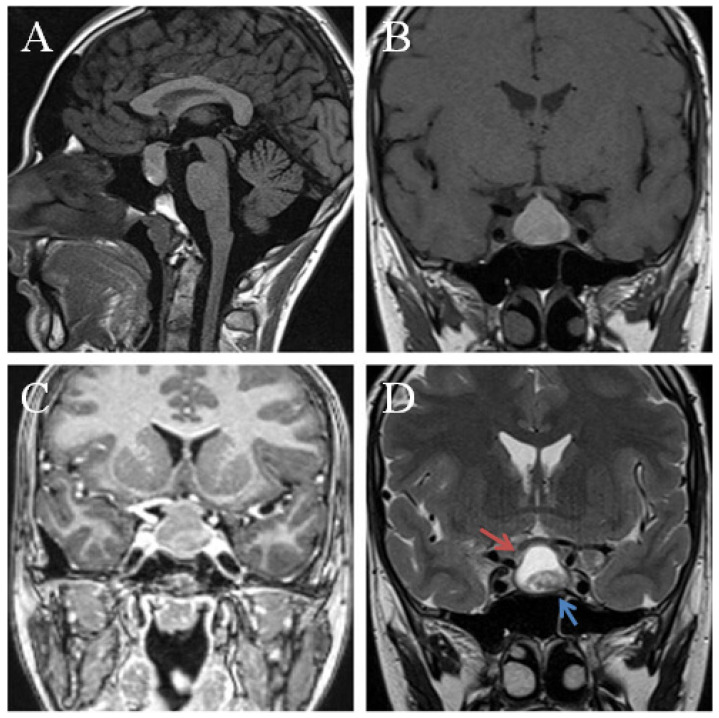
Preoperative MRI of a sellar lesion measuring 25 mm, and postoperatively diagnosed as xanthogranuloma of the sellar region. Sagital pre-contrast T1WI (**A**) demonstrates an isointense signal. The lesion was isointense on T1 precontrast sequences (**B**) with a corresponding mixed signal on T2 sequences (**D**) cystic component hyperintense (red arrow) and a hypointense solid component (blue arrow). Coronal postcontrast T1 (**C**) shows peripheral contrast enhancement. The tumor compressed the pituitary gland and the optic chiasma upward.

The other 16 patients developed mixed tumors on MRI, from which fluid component prevailed in eight subjects and one patient from our experience, illustrated in Figure 3. The solid component dominated in six cases and an equal distribution was evidenced in four cases, respectively. The cystic part shared the same imagistic features with pure cystic XGs, typically hyperintense in T1 and T2 sequences. In predominantly cystic masses, the solid component, represented by fibrosis or chronic hemorrhage, appeared as excentric nodules with low-intensity signal in T2WI, or T1WI and T2WI, and almost no contrast enhancement [15,41,47]. Several heterogeneous solid lesions also presented intratumoral cysts [21].

In light of previous results, in the case series of Fujio et al., the lesions were single-cystic to multicystic in six patients, and solid in three patients [41]. In other case series of Ved et al. and Amano et al., the cystic aspect was noted in all six, and six of seven lesions, respectively [11,22].

### 4.3. Contrast Enhancement of Sellar Xanthogranulomas 

In our review, the majority of XGs (32 of 50) demonstrated no or faint contrast enhancement; however, some other lesions have been contrasted homogenously (4 of 50) or inhomogeneously (10 of 50), respectively. The LR enhancement was noted in nine cases, mainly in cystic lesions with variable content [10,16,26,41,43]. Our data are concordant with previous results provided by Cespedes et al. in a review of 33 sellar and juxta-sellar XGs who underwent MRI of the pituitary gland. Half of the cases showed no contrast enhancement, whereas 30% showed peripheral LR enhancement, and 20% proved heterogeneous enhancement [47]. By contrast, peripheral rim enhancement was identified in seven (50%), and heterogeneous enhancement was detected in 4 of 14 subjects from a study conducted by Yang et al. [5]. Additionally, rim enhancement was present in five of seven cases by Amano et al. [11]. 

### 4.4. Sellar Xanthogranulomas Dimensions, Shape, Location, Epicenter, and Extension to Surrounding Structures

We defined the tumor size as the maximum preoperative tumor diameter recorded. The mean diameter of the included XGs was 25 mm, ranging from 7 to 50 mm.

XGs of the sellar region have been located within both intra- and suprasellar regions in 31 patients (62%), but purely supra- or intrasellar cases have occurred in eight (16%) and one patient (2%), respectively. Five patients (10%) presented, beside the intra- and suprasellar location, an extension through the retrosellar region, third ventricle, right mesial temporal lobe, hypothalamus, foramen of Monro, or tuber cinereum [10,23,39,45,52]. Similarly, Yang et al., in a series of 14 cases from a single-center 10-year experience, showed a 78% prevalence of XGs in intra- and suprasellar locations [5]. 

In line with our results, Hernández-Estrada et al. found that 67% of 27 patients had both intra- and suprasellar locations, 22% had purely suprasellar locations, and only 11% had purely intrasellar positioning [50].

A four-tiered classification of sellar tumor shape, retrieved from Choi et al. [59], was used to categorize XGs as ovoid, snowman-like, or lobulated. Among the 40 of 50 cases for which the tumor shape could be retrieved, the ovoid shape (n = 24) prevailed over snowman-like (n = 9) and lobulated appearance (n = 5). Moreover, the ovoid-shaped XGs were the most common in each subset of lesions divided by pattern of morphology (solid, cystic, or mixed). Interestingly, lobulated masses belonged only to cystic or partially cystic XGs. Choi et al., who focused on the particular imaging features of PAs, CPs, and RCCs, concluded that an ovoid shape is most frequently found in RCCs. By contrast, the snowman-like shape was most common in PA, whereas the superiorly lobulated shape was most typical for CP [59]. Future studies are required to confirm that an ovoid shape is a characteristic feature of XGs, unlike other sellar and para-sellar tumors.

Petrakakis et al. developed and utilized a method of evaluating para-sellar tumor growth pattern and origin [15], dividing the para-sellar region into four quadrants on a midline sagittal T2WI. Two hypothetical lines defined the four quadrants, one connecting the *planum sphenoidale* and the dorsum sellae, and another crossing perpendicularly to the first line where the infundibulum would descend through the *diaphragma sellae* [15]. The author claims that this paradigm enables the comparison of the tumor epicenters, and defines differential growth patterns of sellar and para-sellar masses. The method was further re-used by Ved et al. [22]. In both case series, XGs appear to have suprasellar tumor epicenters corresponding to quadrants 1 and 2. These findings contrast with cystic PAs and RCCs, which typically have an epicenter in sellar quadrants 3 or 4, and support the assumption that XGs originate from the hypothalamic-infundibular region [22]. A primarily sellar tumor epicenter may suggest cystic adenoma or RCC when assessing cystic pituitary lesions, and a suprasellar epicenter indicates a tumor of hypothalamic-infundibular or optic-chiasmatic origin (XG, CP, gliomas, etc.) [22].

Regarding extension to surrounding structures, only 6 of 37 cases with available data demonstrated infiltration of cavernous sinuses. Furthermore, a more extensive case series demonstrated cavernous sinus involvement [15,22]. The involvement of optic chiasm was found more often in the current review (in 28 cases; 56%) compared to four of six cases described by Ved et al. [22]. Remodeling of *sella turcica* manifested as enlarged, caved in, or eroded, and *sella* with irregular margins was seen in 26 cases, whereas normal *sella* appeared in 10 cases. For the other 14 patients, this information could not be retrieved.

### 4.5. Pituitary Gland Appearance on MRI

Of the 19 articles that reported the imaging appearance of the pituitary gland, the majority showed a compressed hypophysis (10 of 19; 52%) [14,20,42,43,46,50,54,56]. In a few cases, hypophysis was separated from the tumor (4 of 19; 21%) [14,52,53,54], or it was absent (6 of 19; 31%) [41,44,47,58]. Only 14 manuscripts provided data about the aspect of the posterior pituitary, of which 10 reported the absence of the bright spot [15,41,42,52,56], and in four cases, the bright spot was visible [15,21,26]. Pituitary stalk appearance could be retrieved from just 19 articles, deviated in 8 and invaded in 10 cases. These imaging features of the pituitary gland and infundibulum are most probably unspecific, and reflect, to a great extent, the tumor dimensions rather than the histological nature of the lesion.

### 4.6. Additional Signs 

Intratumoral calcifications are best depicted with CT (computed tomography), and usually have no signal on spin-echo MRI [60]. It is essential to detect calcification in a sellar lesion because the finding helps restrict the differential diagnosis to CP, aneurysm, chordoma, and cartilaginous tumor [60]. It was previously believed that XGs do not display calcifications on CT scans [13]. Three other recent case series described the absence of calcifications in XGs [5,22,41]. However, in our review, 10 of 29 patients (34.5%) who underwent CT scans displayed intratumoral calcifications. Additionally, a study performed by Amano et al. [11] reported calcifications preoperatively in five of seven cases, and Muller et al. [40] in 38% of 14 pediatric cases, which infirm the initial thesis that sellar XGs do not display calcification. The presence of calcifications in XGs could aid in their differentiation from RCCs. 

An infrequent imaging sign associated with sellar XG was fluid-fluid level within the lesion, found in two cases [10,39]. The authors stated that fluid-fluid level could be generated by the interface between the high signal of cholesterol components and the low signal of granulation and chronic hemorrhage [44]. Hierarchical or signal stratification was described in two cases [26,44], and sedimentation sign was reported by Neubauer et al. in a single case [54]. 

Another particular feature seen by Mohan et al. was the retroclival dural tail and the development of enhancing intra-axial lesions in pons, bilateral middle cerebellar peduncles, and cerebellar hemispheres [52]. The etiology of these signs was unknown; however, the author hypothesized that sellar-suprasellar XG could be a localized manifestation of systemic disease, and the development of intra-axial lesions may represent disease progression [52]. 

In a retrospective study of six XGs, five patients (83%) presented stalk-thickening [22].

### 4.7. MRI Features of Xanthogranuloma in Correlation with Histopathological Diagnosis

There were direct correlations between MRI and histopathological findings in only a few articles. The predominant MRI aspect of the tumors, hyperintense in both T1WI and T2WI sequences without contrast enhancement, appears to be provided by the cholesterol-rich fluid components of the cystic lesions [15,44]. Similarly, hemosiderin cystic fluid appears as T1WI hyper- or isointense and T2WI hyperintense [46]. Areas with high T1WI and low T2WI signals were attributed to cholesterol clefts [21,41,46,58]. Hemosiderin deposits appear as isointense areas in T1WI and low-signal intensity lesions in T2WI scans. Areas of fibrosis or granulation, similar to chronic hemorrhage, appear in both T1WI and T2WI as iso- to hypointense masses with faint or no contrast enhancement [14,41,44,46]. 

The T1WI hyperintensity may indicate cystic content and cholesterol clefts prior to bleeding or lesion degeneration [22]. However, after necrosis or hemorrhage, the lesions may exhibit heterogeneous signals on both T1WI and T2WI, making XGs distinct from other cystic lesions such as RCC and CPs. Calcifications, micro- or localized hemorrhages, and hemosiderin deposits found in tumors can frequently cause heterogeneous cystic appearance, and play an essential role in developing XGs, either as a trigger for granulomatous changes, or as a result of these changes [22,26].

The high signal intensity of XG and other lesions rich in fat-containing compounds is due to the high triglyceride content, which reduces the T1 relaxation time. In the methemoglobin phase of the blood, T1WI provides a high signal. In the late subacute phase of the bleeding, after around seven days, erythrocytes degrade, and extracellular methemoglobin emerges, which shortens the T1 relaxation time and lengthens the T2 relaxation time, making the lesion hyperintense on both T1- and T2WI. After two weeks, methemoglobin is oxidized to hemosiderin, resulting in progressive signal hypointensity on T1- and T2-weighted imaging [61]. 

### 4.8. Imaging Differential Diagnosis of Sellar Xanthogranulomas 

The MRI characteristic features of CPs, RCCs, and pituitary XGs are summarized in Table 2. In our review, from the 31 articles that reported the presumed preoperative diagnosis by imaging findings, nine cases had an unclear morphological pattern and received multiple alternative diagnoses, including RCC, CP, cystic, degenerative, or hemorrhagic adenoma; in Mohan et al., differentials included CPP, meningioma, germinoma, lymphoma, and metastases [52]. In 10 cases, PA was the presumed preoperative diagnosis, from which some were considered hemorrhagic (n = 3), degenerative (n = 3), or cystic macroadenomas (n = 2). CP and RCC were suspected in seven and five patients, respectively. XG was not listed in any patients’ differential diagnoses based on preoperative imaging features. 

RCCs are the most common incidentally found lesions in the sellar area originating from the Rathke’s pouch remnants [62]. They are located between the anterior and posterior pituitary lobes [63]. Imaging parameters that sustain RCC diagnosis are small cyst volume, ovoid shape, and no cyst wall enhancement [59,64]. The absence of enhancement on post-contrast imaging is a shred of significant evidence that these lesions are cystic. On T1WI and T2WI, RCCs have varying signal intensities depending on protein concentration, but they are frequently T1WI hyperintense, and occasionally T1WI iso- or hypointense, with fluctuating T2WI signal. In contrast to XG, enhancement of the cyst wall is rarely and partially present in RCC unaccompanied with inflammation [41]. Moreover, RCCs do not have calcifications [12,65].

Remnants of the cricopharyngeal canal lead to the development of a spectrum of cystic lesions, from simple RCCs to CPs [63]. CPs are divided into two histological subtypes, adamantinomatous CPs (aCPs), which classically occur in children, and squamous-papillary CPs, which primarily affect adults. Imaging features that support a diagnosis of CP include a tumor diameter greater than 2 cm, calcification, and suprasellar location [64,66].

The aCPs manifest as heterogeneous cystic and solid suprasellar multilobulated masses, and up to 90% of them have calcifications on T2WI and CT scans [60,67]. The solid regions have variable signal intensities and enhance contrast [60]. Inhomogenous signals before and after contrast administration are due to small necrotic areas within solid components [60]. The cystic components may display varying signal intensities depending on the protein, cholesterol, or hemorrhage content, but are usually hyperintense on T1-, T2-, and FLAIR-weighted images due to proteinaceous liquid [60]. Papillary CPs appear predominantly as solid lesions with a smooth surface, less commonly calcified, and without a cystic component [68,69,70]. The solid part classically presents isointense signal intensity on T1WI and T2WI. They show homogeneous or reticular enhancement because of the small necrotic areas [60]. 

**Table 2:** Differential diagnosis of sellar XG based on imaging features. Abbreviations: CE = contrast enhancement; CP = craniopharyngioma; CT = computed tomography; F/M = female/male ratio; PA = pituitary adenoma; RCC = Rathke’s cleft cyst; WI = weighted image; XG = xanthogranuloma.

Criteria	Sellar XG	RCC	Adamantinomatous CP	Papillary CP	PA
Mean age (years)	41	38	5–14	65–74	18–73
Sex prevalence (F/M)	1:1	2:1	1:1	1:1	1:1
Component characteristics	Cystic, mixed (solid, cystic portions)	Cystic, intracystic nodules (77%) [63]	Mixed (solid, cystic, lipid components, calcified portions) [60]	Mostly solid or mixed (solid and cystic) [60]	Mostly solid (may contain cystic component or hemorrhage)
Signal intensity on T1WI	Hyperintense cystic component Iso- to hypointense solid component	Hypointense (some are hyperintense)	Hyperintense cystic part [63], variable intensity of the solid part	Isointense solid component	Hypointense [71]
Signal intensity on T2WI	Hyperintense cystic component	Hyperintense	Hyperintense cystic component; Variable intensity of the solid component	Isointense solid component	Variable (usually hyperintense)
Contrast enhancement pattern	No or linear rim CE of the cystic component Hetero- or homogenous CE of the solid component	No or linear rim CE.	Homo- or inhomogeneous (small necrotic areas) CE of the solid component; Peripheral CE.	Homogenous or reticular CE [60]	Homogenous CE [72]
Shape	Round or Ovoid	Round	Multilobulated	Round	Snowman-like
Calcifications on C.T.	In ~34%	-	In ~90% [60]	-	Rare
Main tumor epicenter	Sellar	Sellar	Suprasellar	Suprasellar	Sellar
Tumor extension	Compressing 3rd ventricle	Below optic chiasm [59]	Compressing 3rd ventricle [60]	Compressing 3rd ventricle [60]	Compressing optic chiasm [59]

Uncomplicated, hemorrhagic, or degenerative PAs also fall into the differential diagnoses of sellar XGs. PAs are the most common intrasellar lesions, accounting for 10–15% of all intracranial lesions [73]. Classical imaging findings of an uncomplicated PA include slow contrast enhancement compared with that of the pituitary gland, lateral deviation of the pituitary stalk, and isointense signal on T1WI [73]. Intratumoral hemorrhage and ischemic infarction are frequent in large PAs, resulting in hemorrhagic or cystic changes, leading to various MRI signal intensities [74,75]. The complex appearance of PAs makes them difficult to differentiate from RCC. However, the presence of a fluid-fluid level, septation, and an off-midline location was shown to favor a diagnosis of cystic PA over that of RCC [73]. Conversely, the presence of an intracystic nodule was associated significantly with RCCs [73].

However, because these characteristics are not unique to any of the lesions, there are currently no precise radiological criteria that can reliably distinguish XGs from other cystic sellar lesions. 

The recurrence rate of RCCs and CPs with long-term follow-up is high, ranging from 10 to 40% [76,77]. The current evidence indicates that GTR of the RCC wall has the potential to decrease the incidence of symptomatic RCC recurrences [77]. Similar to RCCs, in adult CPs, GTR is a favorable prognostic factor for recurrence at the expense of the increased risk of postoperative endocrine deficits [76]. Sellar XGs, conversely, show a lower recurrence rate, even after an STR [15]. Moreover, XGs can cause severe persistent hypopituitarism and potentially reversible visual field disturbances [11,13,14,15]. Therefore, a more precise imaging preoperative diagnosis would guide the surgical decision-making towards a more conservative tumor mass reduction with decompression of the optic pathways in highly suggestive cases for XGs. 

## 5. Future Perspectives in Neuroimagistics of Sellar Xanthogranuloma 

As sellar XGs show a variable and nonspecific appearance on contrasted MRI, complementary imagistic modalities are required to improve their presurgical diagnosis. MR spectroscopy (MRS) and diffusion-weighted MR imaging (DWI) are practical noninvasive imaging methods used to characterize different sellar tumors [78]. A case of non-infectious JXG located in the frontal lobe has been described to show the restriction of diffusion on DWI [79]. Moreover, as the features of the most common sellar and suprasellar lesions, such as macroadenoma, CPs, and RCCs with DWI and MRS, have been assessed, [78,80,81] specific analysis of DWI appearances in sellar XG is justifiable. 

Three-dimensional texture analysis is an emerging method to extract quantitative information from imaging modalities in voxel and pixel intensities, and may have a potential role in the discrimination of different tumor types [72,82]. MRI texture analysis has found its purpose in pituitary pathology, being developed to predict the recurrence and response of PAs to medical or surgical treatment. Several MRI texture features have been suggested to be significantly different between PA and CP [72]. In this regard, future studies are expected to improve the preoperative diagnosis of XGs and differentiate them from other tumors of the sellar region. 

## 6. Beyond Imaging Diagnosis: The Molecular Profile of Sellar Xanthogranulomas

The surgical pathology of sellar and juxta-sellar tumors is challenging, and only small tissue specimens are often available for histological evaluation. This issue applies to the differential diagnosis among non-adenomatous tumors, such as CPs, RCCs, or XGs. In recent years, significant progress has been made in identifying sellar non-adenomatous lesions’ molecular profile. Adamantinomatous and papillary CPs harbor clonal mutations that are mutually exclusive [83]. *CTNNB1* gene mutations have been found as molecular markers for aCPs, reported in up to 70% of cases [83,84,85]. The BRAFV600E mutation, conversely, is reported in around 86% of papillary CPs, and has been proposed as a genetic hallmark for these tumors [83]. 

A recent study analyzed 45 non-adenomatous sellar region tumors by target parallel next-generation sequencing, including aCPs, papillary CPs, RCCs, and sellar XGs, integrating molecular and histopathological data [86]. In contrast to CPs and similar to RCCs, all five XGs but one were wild-types for both *BRAF* and *CTNNB1* genes. Interestingly, the identification of mutated *CTNNB1* in one XG sample led to histologic re-evaluation after scrutiny of the deeper histology sections. An aggregate of epithelium with palisade cells compatible with aCP has been found. The diagnosis was revised from XG to aCP [86]. The molecular analysis of these lesions could complement histopathological diagnosis in samples with a limited amount of tissue available for pathological evaluation. Moreover, these data could give insight into the origin and histo-pathogenesis of sellar XGs as a primary lesion or secondary to other cystic lesions. 

Estrada et al. recently described two cases of sellar XGs with negative immunohistochemistry for b-catenin and BRAFV600E [50].

The Wnt signaling pathway could play a role in the tumorigenesis of sellar non-adenomatous masses, and could be potentially used in their molecular differential diagnosis. Under physiological circumstances, the binding of Wnt-protein ligand to a membrane receptor initiates an intracellular cascade that disrupts the destruction of the b-catenin complex, consisting of axin, APC, CK1a, and GSK3b [87]. Consequently, the accumulation of b-catenin into the nucleus, and the interaction with specific transcription factors promote cell proliferation and morphogenesis [88]. The immunohistochemical distribution pattern of b-catenin was examined in an extensive series of 149 different sellar tumors, including PAs, CPs, arachnoid cysts, RCCs, and XGs [89]. The majority (46 out of 49) of aCPs revealed a nuclear expression of b-catenin.

In contrast, all other tumors, papillary CPs, cellular elements of XGs, epithelium of RCs, delicate membranes of arachnoid cysts, and PAs revealed b-catenin immunoreactivity restricted to the cell membrane and cytoplasm [89]. This finding could be helpful in the pathological work-up of surgical specimens to discriminate aCP from other tumors, including sellar XGs. However, additional genetical studies are needed to replicate these results.

## 7. Treatment Options, Long-Term Surveillance, and Outcome

Gross total resection (GTR) is considered the treatment of choice in XG of the sellar region [22]. The endoscopic endonasal approach is preferred over transcranial access [22,26]. However, the transcranial approach is preferred for tumors with considerable lateral or anterior extensions [5]. Considering the low recurrence rate, the favorable neurological outcome, the frequent involvement of the optic pathways, and the poor endocrinological recovery, a mass tumor reduction with decompression of the optic chiasm is regarded as an appropriate surgical goal [15]. In some instances, XGs may occur tightly adherent to adjacent structures; thus, the approved surgical treatment in these cases is maximal safe resection [5,90]. 

Histological confirmation of the XG, considered a benign entity, would avoid unnecessary radiation treatment [14]. Although, radiotherapy was the suitable option for reduction of the mass effect of residual tumors in a case report [91].

The risk of permanent pituitary dysfunction is high. In a case series of 14 XGs, including six patients with pituitary deficits, follow-up examination revealed no substantial change in preoperative status in four cases [5]. In another review of 10 XGs, six of nine subjects with hormonal deficits were followed after surgery, and in five cases, the pituitary function did not recover postoperatively [12]. Moreover, compared to RCC, in XGs and CP, postsurgical endocrine deficiencies were more pronounced, consisting of deficiencies of all four axes [40].

The risk of permanent endocrine deficits depends mainly on the duration between the onset of the symptoms and surgery, reflecting the inflammatory nature of XGs, which cause progressive damage to the normal pituitary tissue [22]. Surgical intervention performed within 3 months after clinical onset or of a non-intrasellar lesion may contribute to the preservation of the pituitary function [26]. The pituitary stalk decompression may lead to an improvement of pituitary function in some cases [41]. Moreover, surgical intervention early in the development of the degenerative changes underlying the XG may improve pituitary function [46]. 

The visual symptoms can be effectively relieved by surgical decompression in the reported series [5,15]. In a review of six XGs, all subjects exhibited significant improvement postoperatively, and no recurrence was recorded over a 56-month follow-up period [15].

The benign and slow-growing character of XGs is confirmed by their favorable outcome and low recurrence rate. In a retrospective review of 295 patients, six with histologically-confirmed XGs reported no recurrence or regrowth after GTR during a median follow-up of 33.5 months [22]. The absence of recurrence or regrowth is rarely mentioned in other cases, even after STR [5,18,42,46,50]. However, the long-term prognosis of sellar XG after surgical resection is yet to be evaluated, and a close clinical and radiological follow-up is needed.

## 8. Conclusions

MRI remains a crucial method for supporting the diagnosis of pituitary lesions and determining the differential diagnosis of various sellar tumors. XG is the ultimate stage of chronic inflammation affecting RCC and, in some cases, aCP, which may have swamped the entire lesion. A totally or partially cystic ovoid-shaped tumor with a sellar epicenter, located within both intra- and suprasellar regions, mainly hyperintense in both T1WI and T2WI sequence, with calcifications, and LR contrast enhancement, without cavernous sinus invasion, constitute a constellation of imaging features that necessitate the consideration of XG in the preoperative differential diagnosis. The predominantly MRI aspect of the tumors, hyperintense in both T1WI and T2WI sequences without contrast enhancement, is provided by the cholesterol-rich fluid or hemosiderin deposits within the cystic lesions. Younger age at presentation, more extended preoperative history, more severe endocrinological deficits, headache, and less severe visual disturbance are clinical features of sellar XGs that may help differentiate them from alternative diagnoses. Besides RCC and CP, hemorrhagic or degenerative Pas should be included in the differential diagnosis of XGs, as suggested by preoperative diagnoses of the cases in our review. As sellar XGs show variable and nonspecific MR features, complementary imagistic modalities, such as MR spectroscopy, diffusion-weighted MR, and texture analysis, are required to refine their presurgical diagnosis. An endoscopic endonasal gross total resection without radiotherapy is the treatment of choice in sellar XGs. However, considering the low rate of recurrence and low chance of endocrinological recovery, mass reduction with decompression of the optic apparatus may represent an appropriate surgical goal. 

## Figures and Tables

**Table 1:** Literature review of the sellar xanthogranulomas imaging features. Abbreviations: A = adenoma; ant. = anterior; CA = cystic adenoma; Ci = caved in; CP = craniopharyngioma; CT = computed tomography; DA = degenerative adenoma; En = enlarged; Er = erodated; F = female; f. Monroe = foramen of Monroe; Ge = germinoma; HA = hemoragic adenoma; H = high intensity; Hetero = heterogeneous intensity; Homo = homogeneous; I = isointensity; inf. = inferior; Inh = inhomogenous; Ir = irregular; IS = intrasellar region; L = low intensity; LR = linear rim enhancement; Ly = lymphoma; M = male; Me = meningioma; Mt = metastasis; MTL = mesial temporal lobe; NA = not available; N = normal; post. = posterior; RCC = Rathke cleft cyst; RS = retrosellar region; post. = posterior; SS = suprasellar region; SSC = suprasellar cistern; sup. = superior; T1WI = T1-weighted; T2WI = T2-weighted; TC = tuber cinereum; “+” = present; “-“= absent; “+/-“= slight/mild/faint; ↓ = diminished; ↑ = significant; 3rd V = third ventricle.

Author, Year, Reference		Tumor Imaging Features						Tumor Location and Extension to Adjacent Structures			Preoperative Diagnosis
	Sex/Age	Maximum Diameter (mm)	T1 W1	T2W1	Contrast Enhancement	Shape	Calcifications on C.T.	Location, Extension	Cavernous Sinus Infiltration	Optic Chiasma Involvement	
Cheng et al., 2021 [20]	M/16	14	I ant., H post part.	L ant., H/L post. part	-	Ovoid	-	IS.	NA	+	
Pilonieta et al., 2020 [43]	F/26	19	Het H	Het H, ↓ density rim	+/-	Inf. lobulated	NA	IS, SS.	-	-	CA/RCC/CP
	F/42	NA	Het H	Het H	LR	Snowman	NA	IS, SS.	NA	+	RCC
	M/35	NA	H	H	LR	Sup. lobulated	NA	IS, SS.	NA	+	RCC/AA
Shao et al., 2020 [44]	F/50	NA	H sup., L inf part	H sup., L inf. part	-	Snowman	NA	IS, SS	NA	+	HA
Fujio et al., 2019 [41]	F/73	NA	H	Het H, ↓ density rim	LR	Ovoid	+	IS, SS.	-	+	NA.
	M/26	NA	NA	L	Inh	Ovoid	NA	SS	-	+	CP
	M/58	NA	H	H sup., L inf. part	NA.	Sup. lobulated	-	IS, SS.	NA	+	NA
	F/54	NA	mainly H, patchy L areas	mainly I to H, patchy L areas	NA.	Irregulated	NA	IS, SS.	NA	NA	NA
Cho et al., 2018 [45]	M/36	46	Het H	Het H	LR	Multi-lobulated	+	SS, RS, 3rd V, MTL	NA	+	CP.
La Rocca et al., 2018 [46]	F/39	14	mainly H, sup. L mass	mainly L, sup. H mass	Inh	Ovoid	NA	IS.	-	-	A
Kobayashi et al., 2018 [42]	F/11	NA	H	H	-	Ovoid	-	IS, SS.	-	+	CP.
Li et al., 2018 [23]	F/56	30	Het I	Het H	↑↑	Ovoid	-	IS, SS, Ht, f. Monroe, 3rd V	NA	+	A
Nishimura et al., 2018 [16]	M/13		mainly H, inf. L mass	mainly H, inf. L mass	LR	Snowman	+	IS, SS.	-	+	CP/RCC/CA
Cespedes et al., 2017 [47]	M/10	7	H	Het I	-	Ovoid	NA	IS, SS.	-	+	CP/A
	M/35	35	H	mainly H, sup. and ant. L area	-	Ovoid	+	IS, SS.	NA	+	NA
	M/31	16	mainly H	NA	+/-	Ovoid	NA	IS, SS.	-	+/-	NA
Dai et al., 2017 [48]	F/36	18	H	H	inh	Snowman	NA	IS, SS.	-	+	RCC
Gurcay et al., 2016 [49]	F/45	NA	Het I	Het I	+	Snowman	-	IS, SS.	+	+	A
Hernandes-Estrada et al., 2016 [50]	F/35	13	Het H	Het H	-	Ovoid	NA	IS.	-	+/-	RCC/CP/CA
	F/32	25	H	H	-	Ovoid	NA	IS, SS.	-	+	CP
	F/40	12	mainly H, central I area	mainly H, central I area	-	Ovoid	+	IS, SS.	-	NA	CP
Petrakakis et al., 2016 [15]	F/66	12	H	H	-	NA.	-	SS.	-	NA.	NA
	M/55	20	H	L	+	NA.	+	SS.	-	NA.	NA
	F/24	17	H	H	-	NA.	-	SS.	-	NA.	NA
	M/28	38	mainly H, inf. L mass	mainly H, inf. L mass	-	Snowman	-	IS, SS.	-	+	NA
	M/29	38	H	H	-	NA.	+	SS.	-	NA.	NA
	M/73	44	H	H	-	NA.	+	IS, SS.	-	NA.	NA
Ji et al., 2016 [51]	F/43	16	Het H	Het H	Inh	Ovoid	NA	IS	-	-	HA
Mohan et al., 2014 [52]	M/37	17	I	mainly I, few L areas	Inh	Ovoid	NA	SS, TC.	NA	+	CP/Me/Ge/Ly/Mt
Agarwal et al., 2012 [53]	M/41		Het H	Het L	Inh	Snowman	+	IS, SS.	-	+	HA
Neubauer et al., 2012 [54]	M/59	27	H	H with dorsal I/ L sedimentation	-	Ovoid	-	IS, SS.	-	+	RCC/CP/CA
Nishiuchi et al., 2012 [26]	M/47	20	mainly I, central H areas	mainly I, central L area	+/-, LR.	Ovoid	NA	IS, SS.	-	-	RCC/A
Miyajima et al., 2011 [1]	M/58	NA	H	H with central L	LR.	Snowman	+	IS, SS.	+	+	NA
Nishioka et al., 2010 [21]	M/33	50	H, I	I, L	Inh	Irregulated	-	IS, SS.	-	NA	DA
	F/50	42	H, I	H, I, L	Inh	NA	-	IS, SS.	+	+	DA
	F/56	14	H, I	H, I	Inh	Ovoid	-	IS, SS.	+	-	RCC
	F/62	31	H, I, L	H, L	Inh	NA	-	IS, SS.	-	NA	CA
	F/67	29	H, I, L	H, L	Inh	Irregulated	-	IS.	+	NA	CA
Arai et al., 2010 [12]	F/55	NA	H	mainly H, patchy L inside	-	Ovoid	-	IS, SS.	-	+	HA/RCC
Sugata et al., 2009 [14]	M/26	30	mainly I, patchy L	L	Inh	Ovoid	-	SS.	NA	+	CP
Pavon et al., 2007 [55]	F/16	NA	mainly H, inf. L mass	H	+	Ovoid	NA	IS.	-	+	NA
Liu et al., 2008 [39]	M/32	42	mainly H, small L area	mainly H, small L area	-	Multi-lobulated	NA	SS, 3rd V, f. Monroe,	+	+	NA
Tajima et al., 2006 [56]	M/9	NA	H	H	-	Ovoid	-	IS.	-	-	RCC
	M/6	NA	H	L	-	Ovoid	-	IS.	-	-	NA
Jung et al., 2005 [57]	F/57	25	mainly H, partially L	mainly H, partially L	+/-	Snowman	NA	IS, SS.	-	+	NA
	NA/5	26	H and L areas	H and L areas	+/-	NA.	NA	IS, SS.	NA	NA	CP
Burt et al., 2003 [10]	M/29	18	Het	Het	LR.	Ovoid	-	IS, SS, SSC.	-	+	NA
	M/26	NA	mainly H, inf L	H, inf. L	-	NA.	NA	IS, SS.	NA	NA	NA
Yonezawa et al., 2003 [58]	M/67	NA	H	mainly L with H areas	-	Ovoid	NA	IS, SS.	-	-	RCC

## Data Availability

Not applicable.
